# Long-Term Stable Organic Photodetectors with Ultra Low Dark Currents for High Detectivity Applications

**DOI:** 10.1038/srep39201

**Published:** 2016-12-22

**Authors:** Marcin Kielar, Olivier Dhez, Gilles Pecastaings, Arnaud Curutchet, Lionel Hirsch

**Affiliations:** 1 University of Bordeaux, IMS, UMR 5218, F-33400 Talence, France and CNRS, IMS, UMR 5218, F-33400 Talence, France; 2 ISORG, 60 Rue des berges, Parc Polytec, Immeuble Tramontane, 38000 Grenoble, France; 3 University of Bordeaux, LCPO, UMR 5629, F-33400 Talence, France

## Abstract

Printed organic photodetectors can transform plastic, paper or glass into smart surfaces. This innovative technology is now growing exponentially due to the strong demand in human-machine interfaces. To date, only niche markets are targeted since organic sensors still present reduced performances in comparison with their inorganic counterparts. Here we demonstrate that it is possible to engineer a state-of-the-art organic photodetector approaching the performances of Si-based photodiodes in terms of dark current, responsivity and detectivity. Only three solution-processed layers and two low-temperature annealing steps are needed to achieve the performance that is significantly better than most of the organic photodetectors reported so far. We also perform a long-term ageing study. Lifetimes of over 14,000 hours under continuous operation are more than promising and demonstrate that organic photodetectors can reach a competitive level of stability for successful commercialization of this new and promising technology.

We have been living in a digital society for nearly two decades. For this reason, the demand for Human Machine Interfaces, i.e. devices used to connect the digital or virtual world with the real world, is growing exponentially^1^,^2^,^3^,^4^,^5^. Sensors based on organic photodiodes are one of the most innovative technologies addressing this market. They can transform plastic, paper or glass into intelligent surfaces making our daily life easier, smarter and more efficient. A few properties set them apart from traditional inorganic electronics. Organic devices can be lightweight, thin, flexible, semi-transparent, wearable and they can be manufactured in large sizes^2^,^6^. By combining carbon-based materials with printed electronics techniques such as spray-coating, stamping, screen-printing, inkjet printing, roll-to-roll processing, it is now possible to produce large-area sensors at a very competitive cost^1^.

Organic photodetectors (OPDs) must meet a list of requirements in order to be integrated in commercial products. The main goal is to convert a light signal into an electric signal. Usually, a negative external voltage is applied to the photodetector in order to enhance charge collection and response time. For a sufficiently large reverse-bias, the photocurrent is independent of the applied voltage and only proportional to the light intensity. The dark current density *J*_*D*_, i.e. the current measured in the device at reverse bias and without light, must be reduced in order to increase the limit of detection (its detectivity) and minimize power consumption. To date, the trade-off between low dark current and high responsivity, i.e. a ratio of the generated photocurrent to incident light power, still remains a real challenge. Figure 1 shows the *state-of-the-art* of organic photodetectors recently reported and compares five important figures of merit. Two important trends can be identified: dark current density has decreased by four orders of magnitude over the last six years and quantum yield has reached a plateau in 65–70% region. That being said, the majority of recently reported OPDs have either a very low (lower than 1 nA cm^−2^ at −2 V) dark current density^7^,^8^, or a relatively high (higher than 60% at −2 V) external quantum efficiency which is directly correlated with the responsivity^9^,^10^,^11^. Also, it is interesting to note that ODPs with very low dark current densities typically present a very small (lower than 1 mm^2^) active area and are used for image arrays^7^,^12^. Along with high responsivity and low dark current density, device architecture should also be optimized by minimizing number of layers, manufacturing steps (such as annealing), and by avoiding power-consuming techniques (e.g. vacuum deposition), to make the whole process compatible with large-area printed technologies and industrial constraints.

It is important to stress that long operational lifetimes of organic photodetectors are required. To date, no systematic study about long-term stability of organic photodetectors has been reported so far in the literature. Thus, it is vital to demonstrate that OPDs can reach a competitive level of stability, exceeding several thousands of hours of continuous operation, for successful commercialization of this new and promising technology.

In this work, we report the conception, fabrication and full characterization of organic photodetectors with active area larger than 2.5 mm^2^ that combine both high responsivity and ultra-low dark current under reverse bias. The band engineering requirements for dark current suppression are illustrated and discussed in details. As a result, we simplified the device architecture as much as possible (e.g. neither hole nor electron interlayers are needed) and used cheaper and thinner active layer materials in contrast to what has recently been reported in the literature. Thus, with only three solution-processed layers and two low-temperature annealing steps, we draw a strategy for efficient photodetectors with a dark current density as low as 0.31 nA cm^−2^, a responsivity of 0.32 A W^−1^ and a detectivity of 3.21 × 10^13^ Jones at −2 V. To the best of our knowledge, we measured the highest linear dynamic range (LDR) reported in the literature for OPDs. More importantly, we also performed a long-term ageing in order to prove high stability of our sensors. Lifetimes over 14,000 hours are observed under accelerated conditions, thus approaching a goal that has been pursued by the industry for over a decade.

## Results

### Materials and design for efficient photodetectors

**Figure 2:**
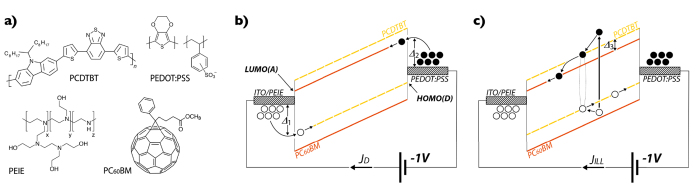
Working principle of organic photodetectors. (**a**) Chemical structures of PCDTBT (electron donor), PEDOT:PSS (top electrode), PEIE (ITO modifier) and PC_60_BM (electron acceptor); (**b**) Working principle of the photodetector in dark illustrating the origin of dark current and (**c**) under illumination showing the photovoltaic effect. Filled circles are electrons, empty circles represent holes.

Chemical structures of the materials used in this work are shown in Fig. 2a. A low bandgap polymer, poly(2,7-carbazole-alt-4,7-dithienyl-2,1,3-benzothiadiazole) (PCDTBT), is used as the electron donor material. PCDTBT, firstly synthetized by Beaupré and Leclerc^27^, has proven over a few years to be a promising candidate for photovoltaic cells with power conversion efficiencies (PCE) close to 7% and internal quantum efficiencies (IQE) approaching 100%^28^. Picking up an efficient polymer is essential for high responsivity photodetectors. PCDTBT is usually mixed with [6,6]-phenyl-C71-butyric acid methyl ester (PC_70_BM) as an electron acceptor in order to achieve an efficient bulk heterojunction (BHJ).

PC_70_BM slightly enhances the light absorption in the visible region (from 400 to 500 nm) and thus the overall efficiency of organic solar cells^27^. Since our photodetectors operate mostly under green, yellow and orange light (500–600 nm), we decided to choose a [6,6]-phenyl-C61-butryic acid methyl ester (PC_60_BM) as an electron acceptor, which is not only significantly cheaper than PC_70_BM but also fits better energetic requirements for dark current suppression, discussed further. PCDTBT and PC_60_BM represent a good model BHJ allowing large open circuit voltage *V*_*OC*_, efficient electron transfer and charge separation at the interface. More importantly, a good stability of this π-conjugated system has also been reported^27^,^29^. We stress the importance of stability as we process our devices in air and under illumination which may lead to photo-induced oxidation^29^,^30^. To minimize this effect, we processed our OPDs under extremely weak (<50 lumens) monochromatic red light (636 nm), taking advantage of low absorption of PCDTBT in the 625–800 nm region. Another striking feature of PCDTBT:PC_60_BM is that neither thermal annealing nor additives were needed to achieve photodetectors with high responsivity. Similar results have been previously reported in the case of organic solar cells^27^,^28^, which makes the processing of the active layer rapid and straightforward.

Polyethylenimine ethoxylated (PEIE) is used to adjust the indium tin oxide (ITO) bottom electrode workfunction (WF). This polymer contains simple aliphatic amine groups that are physisorbed onto the ITO surface. The intrinsic molecular dipole moments associated with these groups and the charge-transfer nature of their interaction with the surface, were found by Kippelen *et al*. to reduce the WF of many conductors^31^. More precisely, a thin layer of PEIE has been reported to lower the WF of ITO by up to 1.0 eV, when spin-coated from 2-methoxyethanol diluted solution^12^,^23^. Due to the toxicity of 2-methoxyethanol, we decided to use deionized water (DI-H_2_O) as solvent to spin-coat PEIE solution on ITO. We found that the WF of ITO decreases from 5.1 to 4.1 eV, when the concentration of PEIE in water increases up to 0.60 wt.%, as presented in Table 1.

**Figure 3:**
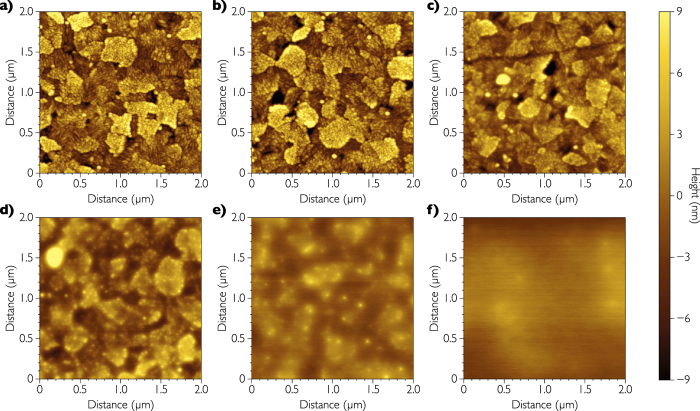
Morphology of the transparent electrode. AFM images of (**a**) pure ITO, and of PEIE films spin-coated from different concentrations in water: (**b**) 0.075%, (**c**) 0.15%, (**d**) 0.30%, (**e**) 0.45% and (**f**) 0.60%.

Having a large offset between the electrode Fermi level and the semiconductor HOMO level has been reported to be a key element to minimize hole injection and thus reduce the dark current^2^. The HOMO level of PCDTBT is located at 5.5 eV and is one of the deepest levels recorded in the literature for photovoltaic polymers^27^. In consequence, the offset between the WF of ITO/PEIE (electrode Fermi level) and the HOMO level of PCDTBT is as high as 1.4 eV. The best concentration of PEIE, at which dark current is extremely low, is optimized to be 0.45 wt.% in H_2_O. The low dark current values confirm a full PEIE surface coverage on ITO. At higher concentrations, we still observe low dark current values but the thicker PEIE layer increases the contact resistance that prevents photo-generated charge extraction^32^. In contrast, for lower concentrations our results show that despite low values of WF, higher dark current densities are observed. It probably results from a non-fully PEIE surface coverage on ITO. We thus performed AFM observation of pure cleaned ITO and ITO/PEIE films for different concentrations of PEIE solution. AFM images are presented in Fig. 3. One can observe that the surface roughness decreases with the increase of the PEIE thickness. These results also highlight that we have to obtain a full PEIE coverage of ITO (Fig. 3e) to achieve the best OPD performances. This thickness corresponds to a roughness (*RMS*) of 1.52 nm, which is significantly lower than for a pure ITO film (3.78 nm). If the PEIE film is too thick (Fig. 3f), charge collection is reduced because PEIE is an insulator.

For the deposition of the top, high WF electrode by screen-printing, we selected a conductive ink formulation based on poly(3,4-ethylenedioxy-thiophene):poly(styrenesulfonate) (PEDOT:PSS). PEDOT:PSS is a well-known and widely used hole selective material^33^. In order to get high conductive top electrode, we screen-printed a relatively thick PEDOT:PSS (1.55 μm) to obtain a sheet resistance of about 20 Ω sq^−1^ almost as low as ITO (10 Ω sq^−1^ for 110 nm). The work function of PEDOT:PSS is measured by Kelvin probe and equals to 5.01 ± 0.07 eV, which leads to a built-in potential of 0.9 V with reference to ITO/PEIE bottom electrode and a barrier of 1.3 eV with regard to the LUMO level of PC_60_BM. We specifically selected the PC_60_BM instead of PC_70_BM to reduce the dark current since the electron injection barrier is larger (1.3 eV vs. 0.7 eV)^34^. Photodetectors with PC_70_BM for similar thickness and bias have reported dark currents reported slightly higher^11^,^12^. Actually, an ultra-low dark current level is crucial and determines the limit of detection of photodetectors. Within this device architecture, both hole and electron injection barriers at reverse bias, Δ_1_ and Δ_2_ respectively, as shown in Fig. 2b, are significant (1.4 eV and 1.3 eV) and lead to an extremely low dark current density of 0.31 nA cm^−2^ at −2 V. Charge collection of photogenerated carriers is efficiently driven by both the internal built-in potential and the external negative bias, as illustrated in Fig. 2c. Our results imply that the key element is to carefully select the materials with regard to their energetic levels, and additional interlayers such as MoO_3_^35^, poly-TPD^25^, or PVK^36^ are not always required. Reducing the number of layers makes the fabrication process easier, faster and cheaper. This is also a requirement for the industrialization of this technology.

### Current-voltage characteristics

**Figure 4:**
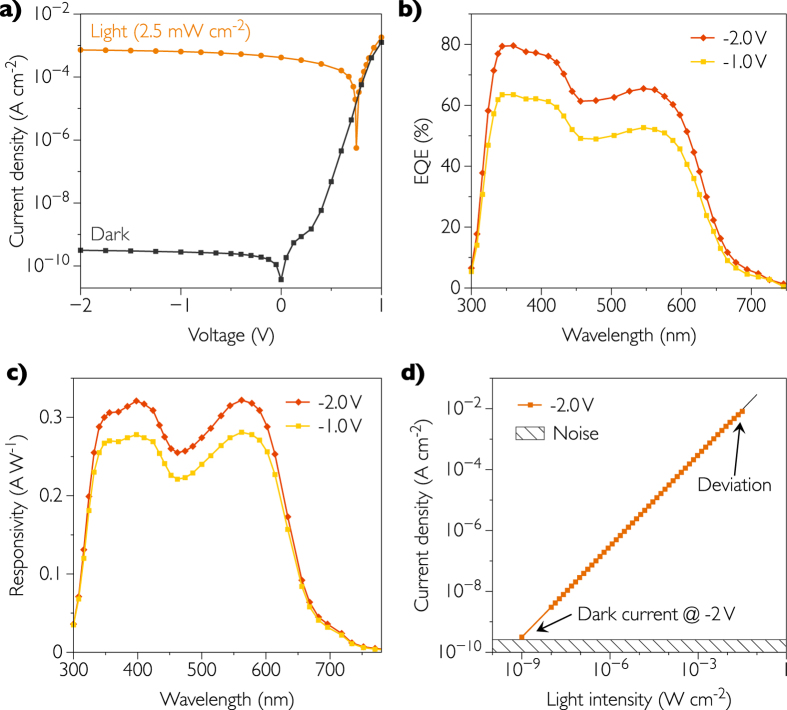
Performance of organic photodetectors. (**a**) Current-voltage characteristics of the optimized device under 528 nm green light. The intensity of monochromatic light is set to 2.5 mW cm^−2^. (**b**) Measured EQE spectral response of OPDs at −1 and −2 V. (**c**) Calculated responsivity spectra of the photodetector from the EQE measurement. (**d**) Photocurrent density as a function of incident power.

Figure 4a shows the current density-voltage (J-V) characteristics of the optimized device in dark and under illumination. A high power LED (528 nm green light) has been calibrated with an integrated sphere to adjust monochromatic light intensity from 10 nW cm^−2^ up to 100 mW cm^−2^. For J-V measurements, light intensity has been set at 2.5 mW cm^−2^. The dark current density of the device is as low as 0.3 nA cm^−2^ at −2 V. The photocurrent density at the same bias reaches a value of 0.75 mA cm^−2^. Thus, the on/off ratio is as high as 6 orders of magnitude. From these results, we can calculate the responsivity, which is expressed as


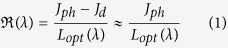


where *J*_*ph*_ is the current density under illumination, in mA cm^−2^, *L*_*opt*_ is the incident light power density, in mW cm^−2^, and *J*_*d*_ is the dark current density which is extremely small as compared to *J*_*ph*_ and therefore can be neglected. The responsivity is equal to 0.3 A W^−1^ at −2 V and 528 nm. In contrast, at −1 and 0 V, the responsivity drops to 0.27 and 0.17 A W^−1^ respectively emphasizing the importance of reverse bias in order to collect holes and electrons at electrodes. Reverse bias of −2 V is found to be the best compromise between a low dark current and a high responsivity since the photocarriers collection reaches a plateau in this region. These results demonstrate that organic photodetectors can compete with inorganic photodetectors such as Si, as the corresponding dark and light levels are very close at reverse bias.

### Spectral shape of the EQE and responsivity

External Quantum Efficiency (EQE) is another important figure of merit as it defines the ratio between the number of photogenerated electrons flowing in the external circuit and the number of incident photons at a given wavelength. Figure 4b shows the EQE spectra of our inverted-structure OPDs at a bias of −1 and −2 V. According to this measurement, the device ranges from 325 to 625 nm covering almost the whole visible spectrum. EQE values exceed 65% at −2 V for 528 nm which corresponds to the wavelength of the LED used in this work as excitation source. The active layer thickness is a key for high EQE values. The majority of the reported OPDs have very thick (from 400 nm to 4 μm) active layers as the thick layer is assumed to reduce the dark current density^2^,^7^,^11^,^21^.

However, a thick active layer also affects the photogenerated current as a part of photocarriers recombine before reaching the electrodes^37^. To overcome this problem, we keep the active layer thickness to be as low as 220 nm. A thinner active layer would result in higher dark current densities (exceeding 1 nA cm^−2^ at −2 V) whereas having a thicker active layer would reduce the quantum yield (below 65% at −2 V). Figure 4c shows the responsivity ℜ calculated the from the EQE spectra, using the following equation:


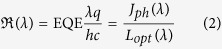


The responsivity reaches the maximum of 0.32 A W^−1^ at −2 V and 566 nm. A responsivity of 0.3 A W^−1^ is calculated at −2 V and 528 nm and perfectly matches the results we obtained with the high power LED. We would like to emphasize that these values are comparable with those obtained with a Si photodetector in the same conditions.

### Linearity measurements

The linear dynamic range (LDR) corresponds to the proportionality of the photocurrent with respect to the optical power. Despite the fact that the definition of LDR is quite simple, one can find many discrepancies in the methodology reported in the literature, which is somewhat surprising and meaningless. In general, the LDR can be expressed as:


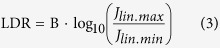


where B is a constant, *J*_*lin.max*_ and *J*_*min.lin*_ are the maximum and minimum values of the linear portion of the curve, respectively. The unit of LDR is decibel (dB). *J*_*lin.max*_ is commonly named “deviation current” and expressed as 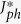 in all reported OPDs. Nonetheless, there is no rigorous method for evaluating this current. The main problem comes from the definition of *J*_*min.lin*_. It is reported as the dark current density *J*_*D*_ at a given reverse bias^1^,^13^,^16^,^20^,^25^,^37^,^38^, or as the theoretical noise current density *J*_*N*_, discussed further^15^. One can also find *J*_*min.lin*_ defined as the dark current density at “0 V” bias which might correspond to the noise current of the experimental setup^8^,^9^,^11^. The constant B is reported to be either 20^1^,^8^,^9^,^11^,^13^,^16^,^20^,^25^,^37^,^38^ or 10^15^,^35^,^39^,^40^. From such different and not clear ways of determining LDR, it is evident that comparing OPDs can lead to ambiguous conclusions since the values can differ from 50 to 240 dB, depending on the method and the photodetector. To address this issue, we define the LDR as shown in equation (4), which is, in our opinion, the most straightforward, comprehensible and consequential method.


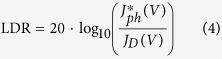


The decibel unit is typically used in electrical circuits to define a power gain. Because power is proportional to the square of current, we set B = 20. 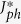
(V) is the photocurrent at a given reverse bias V for which the gain drops 1 dB below the linear, ideal signal called *J*_*id*_. *J*_*D*_(V) is the dark current measured at the same bias. From equation (4) we can determine a 1 dB drop as the point which satisfies the relation 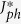 = 0.891 × *J*_*id*_. From our measurements, presented in Fig. 4d, 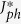 is determined for an illumination of 31.7 mW cm^−2^ and reaches a value of 8.14 mA cm^−2^ at −2 V. *J*_*D*_ is equal to 0.31 nA cm^−2^ at the same bias which corresponds to the minimum detectable irradiance of 1 nW cm^−2^. Therefore, the LDR is 148 dB at −2 V corresponding to the linearity over 7 orders of magnitude. When compared to others reported OPDs, by using the same method, we obtained the highest value of LDR reported to date for OPDs. In contrast, Si detectors still have higher LDR values (from 220 to 240 dB)^9^,^11^,^13^, whereas our OPDs can compete with GaN (100 dB)^40^ or InGaAs (132 dB)^13^ photodetectors. Deviation from linearity at higher light intensities is assigned with high bimolecular recombination rate previously reported for PCDTBT polymer^37^. Hence, the higher the incident power, the larger reverse bias is required to enhance the electric field and fully extract photocarriers. On the contrary, higher dark currents are expected when increasing reverse bias, which degrades the limit of detection. In our case, a reverse bias of −2 V is found to be the best compromise between low dark current, high responsivity and high LDR.

### Transient photocurrent response characterization

**Figure 5:**
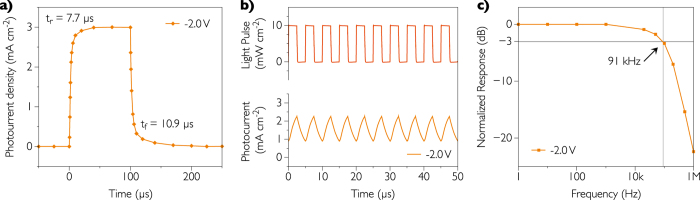
Transient photocurrent response of the photodetector. (**a**) Photocurrent response to a 100 μs light pulse at 528 nm with an incident power density equal to 10 mW cm^−2^. (**b**) Transient photocurrent response at a pulse frequency above the cut-off frequency (200 kHz). (**c**) Measured cut-off frequency (f_−3dB_) in Hz at −2 V reverse bias.

Figure 5a displays the photocurrent response to a 100 μs light pulse with an incident power density of 10 mW cm^−2^ at 528 nm. The photodetector is biased at −2 V and exhibits a rise time *t*_*r*_ (time for which device response rises from 10 to 90%) and a fall time *t*_*f*_ (time for which sensor response decreases from 90 to 10%) of 7.7 and 10.9 μs respectively. The rise and fall times are one order of magnitude faster than in the case of fully-inkjet-printed organic photodetectors with the well-known P3HT:PC_61_BM blend^10^, and one order of magnitude slower than for organic photodetectors with PDPP3T:PC_71_BM blend having the metallic electrode evaporated under high vacuum^25^. The relatively long response of devices indicates the slow extraction process of photogenerated charge carriers, solution-processed electrodes (here PEDOT:PSS) being less conductive than metal electrodes deposited under high vacuum (Ag, Au, Al). We note that this behavior is directly correlated with the series resistance of the photodetector, discussed further. We also observe that photodetectors with fast response times have dark current levels relatively high^11^,^18^,^23^,^35^, which confirms that hole or electron blocking layers may also affect photocarriers collection.

To further explore the dynamic behavior of our OPDs, we measured their frequency response for the light pulse of 10 mW cm^−2^, as illustrated in Fig. 5b (200 kHz) and 4c (from 1 Hz to 1 MHz). The important parameter is the cut-off frequency *f*_−*3dB*_, i.e. the frequency at which the input signal power (and not the current) drops by 50%, which corresponds to a gain of −3 dB. This means that at the cut-off frequency, the measured current *J*_*ph*_ decreased to 0.71 (and not 0.5) of its initial value *J*_*0*_ at the lowest frequency, as described in the following equation:


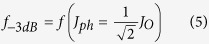


We stress the importance of this definition, which can be also easily demonstrated from equation (4). The cut-off frequency in our case was found to be 91 kHz, mostly independent on the reverse bias used. At higher frequencies, our devices show a linear decreasing response up to 9 MHz. Obviously, we could shift frequency cut-off toward high frequency by reducing the area of OPD and series resistance. Frequency cut-off is directly related to the RC time constant of the equivalent circuit where R and C are the resistance and the capacitance of the device respectively. The series resistance of electrodes is measured at forward bias and the capacitance in the region independent of the frequency (up to 100 kHz). We found R = 3.7 kΩ and C = 450 pF. Thus, the resulting equivalent cut-off frequency is 95 kHz, which is close to the value measured. One can note that this frequency is higher than for fully solution-processed^10^,^12^, non-polymeric^23^ or fullerene-free^9^ OPDs recently reported. Nevertheless, it is difficult to compare cut-off frequencies because they depend not only on the materials but also on the device area, electrode resistivity and light intensity^10^.

### Specific detectivity

**Figure 6:**
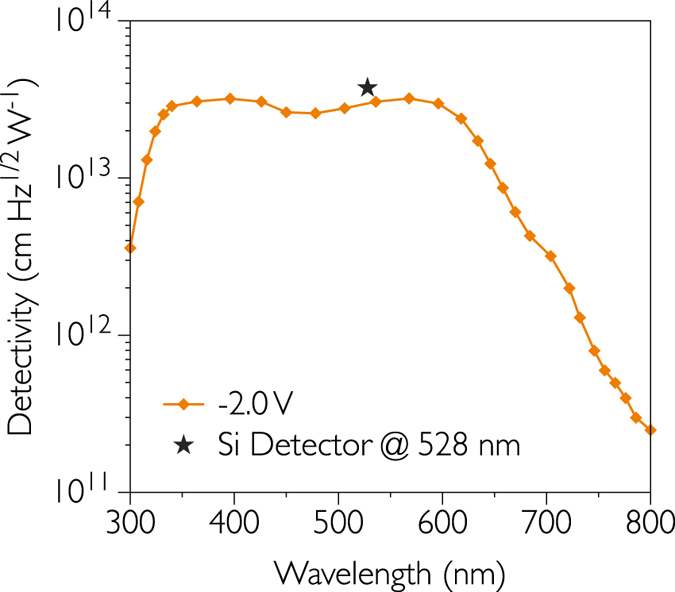
Detectivity of the optimized organic photodetector. Calculated detectivity spectra for the inverted structure photodetector at −2 V. An inorganic Si photodetector is shown for comparison.

To characterize the limit of detection of our devices, another figure of merit called detectivity D^*^ is frequently reported. The detectivity can be given by the following expression:


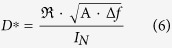


where ℜ is the responsivity in A W^−1^, *A* is the photodetector active area in cm^2^, *Δf* is the electrical bandwidth in Hz, and *I*_*N*_ is the noise current in A. The unit of D^*^ is cm Hz^1/2^ W^−1^ or Jones. Many mechanisms can generate the noise current inside organic devices, only three of them are usually cited: shot noise generated by the random fluctuations in the normal current flow through the device, Johnson noise from the random thermal motion of electrons and flicker noise (or 1/*f* noise) which is not yet fully understood^41^. The shot noise and Johnson noise are white noise, i.e. frequency independent, while the flicker noise is inversely proportional to the frequency. We have to point out that in the topic of electronic noise in organic sensors no seminal studies can be found in the literature. In the case of organic photodetectors in which reverse bias is applied, the shot noise from the dark current is commonly assumed to be the dominant contribution^2^,^13^,^14^,^42^, thermal and flicker noise being “negligible”. If so, the specific detectivity can be expressed as:


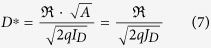


where *q* is elementary charge, *I*_*D*_ is the dark current in A and *J*_*D*_ is the dark current density in A cm^−2^. In our case, the specific detectivity is calculated to be 3.21 × 10^13^ cm Hz^1/2^ W^−1^ at 566 nm and −2 V, as shown in Fig. 6. At −1 V, the detectivity is calculated to be 2.98 × 10^13^ Jones. These values are one of the highest reported in literature.

For comparison, we have calculated in the same conditions the detectivity of a Si detector. It has been determined at 3.75 × 10^13^ Jones, which is very close to our organic sensors. We note that all organic and inorganic photodetectors are a few orders of magnitude below the detectivity of the dark adapted human eye (~10^17^ Jones)^43^.

### Long-term ageing

**Figure 7:**
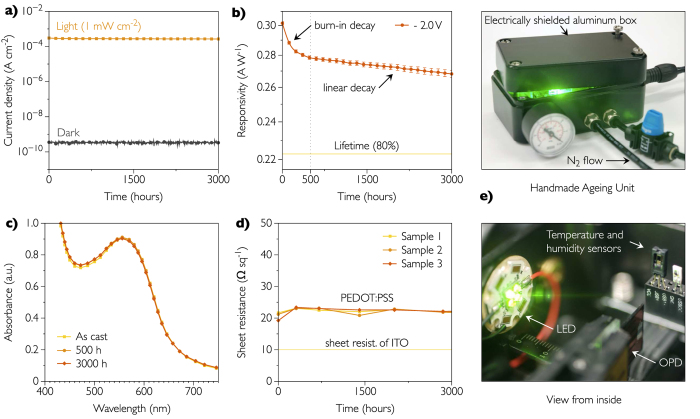
Aging study of organic photodetectors. (**a**) Light and dark current densities of the photodetector illuminated and biased continuously at −2 V as a function of time. In average, a dark current measurement occurs every 30 min. (**b**) Responsivity decay at −2 V with time. (**c**) UV-Vis analysis of the PCDTBT:PC_60_BM as a function of time. Films are continuously illuminated at 1 mW cm^−2^. (**d**) Sheet resistance changes of PEDOT:PSS with time. Films are continuously illuminated at 1 mW cm^−2^. (**e**) Photographs of the handmade ageing unit.

In order to reduce noise coming from environment, the organic photodetectors were kept in a Faraday cage (an electrically shielded aluminum box) during the ageing. The handmade ageing setup can be seen in Fig. 7. A constant nitrogen flow of 100 mL min^−1^ was maintained inside the box to protect materials from environmental factors and minimize the availability of oxygen and humidity. In other words, the devices stored inside the nitrogen-filled box can be regarded as well encapsulated. Our sensors faced with a worse-case scenario since they were constantly biased at −2 V and illuminated. The light was switched off shortly (3 seconds) for dark current measurement and the time delay between two measurements was increasing exponentially. In average, one dark and light readings were programmed every 30 min of constant illumination. For an accelerated measurement, the light intensity was set to 1 mW cm^−2^ which allows to keep the on/off current ratio close to 10^6^, as shown in Fig. 7a. We would like to stress that no ageing studies concerning photodetectors can be found in the literature and, thus, there is no experimental protocols available. For the majority of commercial applications, the on/off current of about 10^4^ or even lower is acceptable as this would reduce power consumption of the setup. Using a high intensity light source (here 1 mW cm^−2^) allows to operate and interpret accelerated ageing experiments for organic photodetectors to determine their lifetimes quickly.

An initial decay of the responsivity that corresponds to the burn-in, was observed during the first 500 h, as shown in Fig. 7b. This degradation has been widely studied in the case of organic photovoltaics and can be attributed to a photochemical reaction in the photoactive layer which creates states in the bandgap of PCDTBT^29^. The burn-in drop mainly concerns *V*_*oc*_ and *FF* (fill factor), and the photocurrent is reported to be only slightly affected. This behavior was also observed in the case of PCDTBT:PC_60_BM^44^. In our case, the drop in photocurrent is relatively low (6%) and corresponds almost to the burn-in observed in Fig. 7b.

A reorganization of PCDTBT films due to the modification of the 2,7-carbazole building block under illumination has also been reported to explain the burn-in^45^. The responsivity drops by 9% during the burn-in period. After nearly 500 h, the exponential loss stops and a linear degradation begins. The slope of this linear portion determines the photodetector lifetime, which is chosen to be the time for the photodetector to reach 80% of post-burn-in responsivity. The average slope of the post-burn-in efficiency loss-rate is measured to be 3.93 × 10^−6^ A W^−1^ h^−1^ leading to the lifetime of 14,400 hours at 1 mW cm^−2^. We keep in mind that this is an accelerating ageing study and, for lower irradiances, the corresponding lifetimes would be even higher than 14,400 hours. This lifetime is very promising given the fact that the operating temperature of the photodetector is only 25 °C for the majority of applications (detection of objects, medical imaging, human-machine interfaces). This is in contrast to organic solar cells where the temperature of the device can easily reach 85 °C which is reported to deteriorate the device performances even without light^46^.

In parallel with ageing study, we monitored UV-Vis spectra of the films PCDTBT:PC_60_BM illuminated at 1 mW cm^−2^, as illustrated in Fig. 7c. A very slight absorption evolution in the first 500 h suggests a rearrangement of the active layer but no polymer degradation is observed at this stage. After 3,000 h, we only observe a 1.11% drop and a 1.5 nm blue-shift of the absorption peak of PCDTBT. The sheet resistance of PEDOT:PSS was also studied over 3,000 h because any degradation of the top electrode would immediately impact its conductivity and result in increased sheet resistance. From our measurements presented in Fig. 7d, no significant change is observed and the sheet resistance is measured at 22 Ω sq^−1^ leading to the conclusion that PEDOT:PSS is stable under strong green illumination. One of the reasons of this high stability comes from the fact that the different suppliers of PEDOT:PSS employ unspecified additives that are present and that increase its longevity.

## Discussion

The above results pave the way for the development of photodetectors with *state-of-the-art* performances in terms of dark current, quantum yield and stability. Energetic levels requirements are the key elements to lower the dark current. In this work, we have demonstrated that one can achieve extremely low dark current and high detectivity with only three solution-processed layers and two low-temperature annealing steps. Our results demonstrate that organic photodetectors can compete with their inorganic counterparts for applications that do not require high cut-off frequencies. Technological process to manufacture OPDs is quite simple and with a careful optimization of all fabrication steps, including material formulation and screen-printing, we have been able to obtain long-term stable organic photodetectors. Lifetimes of over 14,000 hours under continuous operation are encouraging and demonstrate that printed photodetectors can reach a competitive level of stability for successful commercialization.

## Methods

### Materials

PCDTBT (Mw = 110 kDa) was purchased from *St-Jean Photochimie* (SJPC) Inc. PC_60_BM (>99.5%) was purchased from *Solaris Chem Inc.* PEDOT:PSS conductive screen printable ink (5.0 wt.%) and PEIE (80% ethoxylated solution, 35–40 wt.% in H_2_O) were purchased from *Sigma-Aldrich Corp*. All materials were used without any further purification.

### Sample preparation

The indium tin oxide coated glass substrate (10 Ω per square, *Visiontek*) was sequentially cleaned in an ultrasonic bath with acetone, ethanol and isopropanol (15 min each) before UV-ozone treatment (10 min). PEIE solution (35–40 wt.% in H_2_O) was further diluted to 0.075, 0.15, 0.30, 0.45 and 0.60 wt.% in deionized water, then spin-coated at 5,000 rpm for 60 s on the ITO substrates, samples were then dried at 100 °C for 10 min in air. PCDTBT with PC_60_BM were mixed at 1:3.5 weight ratio with overall concentration of 45 mg mL^−1^ in 1,2-dichlorobenzene (99.9%, anhydrous, *Sigma Aldrich*). The solution was stirred overnight at 80 °C in sealed vial. The active layer was deposited by spin-coating in a N_2_-filled glovebox environment (O_2_ and H_2_O < 0.1 ppm) at 700 rpm for 180 s to achieve a thickness of 220 nm. The samples were then moved to a cleanroom in order to screen-print PEDOT:PSS through an ultra-thin metal mask, and annealed in an air-filled oven at 90 °C for 15 min. The screen-printing machine was the *Hydrosilk* supplied by *Schmid Machines SA*. As the active layer may be sensitive to the light exposure in air, the whole process was carried out under very low red light intensity (<50 lumens) in the cleanroom. The final thickness of the top electrode was measured to be 1.55 μm. All devices were then tested under nitrogen flow.

### Device Characterization

The responsivity, the current-voltage characteristics and the linearity measurements were recorded by using a high power, green (528 nm) LED supplied by *Intelligent LED Solutions*, calibrated with a 6-in. diameter calibrated integrated sphere (*Labsphere*), and double checked with a silicon diode (*Centronic Ltd*). A *Keithley* 2604B dual channel source measure unit was used to power the LED, bias the photodetector and record data. All devices were shielded in a metal box (Faraday cage). For the quantum efficiency measurements, a lab-made setup built with a 500 W Xe lamp and Triax 180 monochromator supplied by *Horiba Scientific* and a *Keithley* 6487 picoammeter was used. The incident light power was calibrated with the integrated sphere. Film thickness was measured with *KLA Tencor Alpha-Step IQ* surface profilometer. UV-Vis absorption spectra was recorded with *SAFAS UVmc* spectrometer. A *Tektronix* DPO4104B-L oscilloscope and a *Keithley* 3390 waveform generator were used for frequency and transient measurements. The electrode workfunction were measured with a *Besocke Delta Phi* Kelvin probe 07 control unit calibrated with highly ordered pyrolytic graphic (HOPG) freshly cleaved. Sheet resistance measurements were carried out with *Lucas Labs* four point probe and with a *Keithley* 2400 source measure meter. Noise measurements were recorded by using a *Signal Recovery* M5182 current preamplifier and a *HP* 3562A dynamic signal analyzer. AFM images were obtained with *Bruker Innova* large area scanner atomic force microscope.

## Additional Information

**How to cite this article:** Kielar, M. *et al*. Long-Term Stable Organic Photodetectors with Ultra Low Dark Currents for High Detectivity Applications. *Sci. Rep.* **6**, 39201; doi: 10.1038/srep39201 (2016).

**Publisher's note:** Springer Nature remains neutral with regard to jurisdictional claims in published maps and institutional affiliations.

## Supplementary Material

Supplementary Information

## Figures and Tables

**Figure 1:**
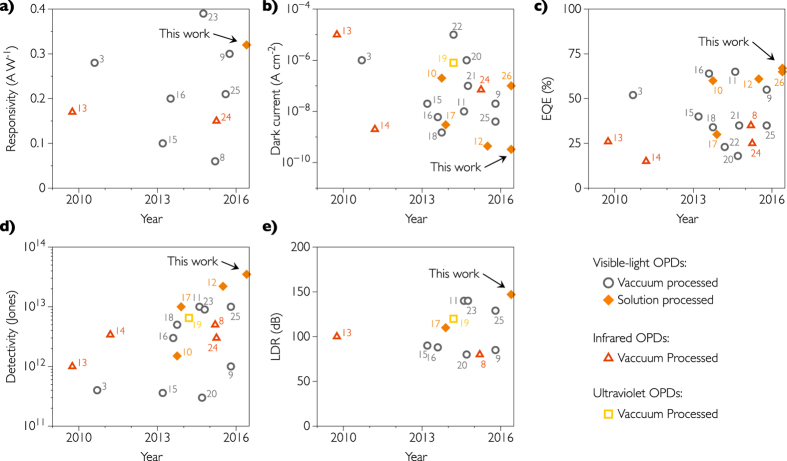
The state-of-the-art of organic photodetectors. Comparison of **(a)** responsivity, **(b)** dark current density, **(c)** external quantum efficiency, **(d)** specific detectivity and **(e)** linear dynamic range for organic photodetectors reported recently. Where it is possible, the value is given at −2 V reverse bias. Plain numbers indicate references^3^,^8^,^9^,^10^,^11^,^12^,^13^,^14^,^15^,^16^,^17^,^18^,^19^,^20^,^21^,^22^,^23^,^24^,^25^,^26^.

**Table 1:** Dark current suppression.

PEIE concentration [wt.%, in H_2_O]	PEIE roughness (RMS) [nm]	Work function of ITO [eV]	Dark current [A cm^−2^, −2 V]
No PEIE	3.78 (±2.72)	5.11 (±0.07)	7.6 × 10^−6^ (±2.3 × 10^−6^)
0.075	3.50 (±2.42)	4.25 (±0.05)	6.2 × 10^−8^ (±1.9 × 10^−8^)
0.15	3.01 (±2.17)	4.21 (±0.10)	6.0 × 10^−9^ (±2.8 × 10^−9^)
0.30	2.72 (±1.90)	4.16 (±0.04)	5.3 × 10^−10^ (±1.7 × 10^−10^)
0.45	1.52 (±1.12)	4.12 (±0.05)	3.1 × 10^−10^ (±9.3 × 10^−11^)
0.60	1.01 (±0.51)	4.12 (±0.07)	2.7 × 10^−10^ (±1.1 × 10^−10^)

Roughness of a PEIE layer measured by AFM, workfunction of ITO measured by Kelvin probe and dark current density of the fabricated sensors at −2 V as a function of PEIE concentration in deionized water. Standard deviations are indicated in brackets.
