# Supplementary material for: Long-Term Stable Organic Photodetectors with Ultra Low Dark Currents for High Detectivity Applications
Source: Sci Rep. 2016 Dec 22;6:39201. doi: 10.1038/srep39201 (PMC5177896; doi:10.1038/srep39201)
Supplement: Supplementary Information [file srep39201-s1.pdf]

Long-Term Stable Organic Photodetectors with Ultra Low Dark Currents for High Detectivity Applications.

Marcin Kielar, Olivier Dhez, Gilles Pecastaings, Arnaud Curutchet and Lionel Hirsch*

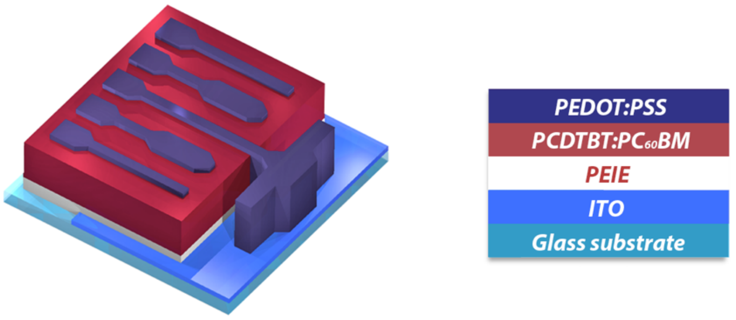

Supplementary Fig. 1 | Device design and structure. Four photodetectors with an inverted structure ITO/PEIE/PCDTBT:PC_{60}BM/PEDOT:PSS are fabricated on a 15x15 mm^2 glass substrate. Two of them have the active area of 2.77 mm^2, the others two 5.53 mm^2. The common electrode for 4 devices, to get contact with ITO, is located in the center.

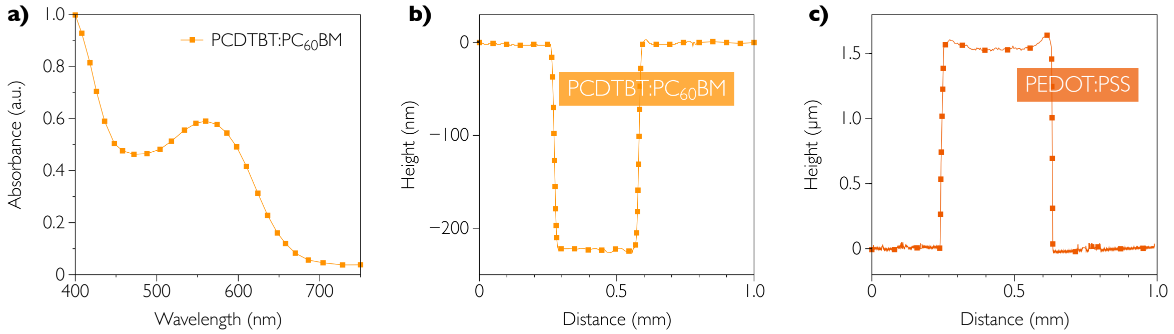

Supplementary Fig. 2 | Organic layer properties. (a) Absorption spectra of the film PCDTBT:PC_{60}BM. Profilometer measurement of **(b)** spin-coated PCDTBT:PC_{60}BM active layer (220 nm) and **(c)** screen-printed PEDOT:PSS (1550 nm).

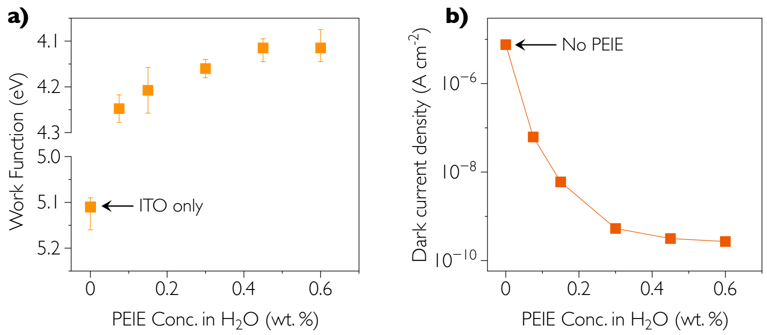

Supplementary Fig. 3 | Dark current suppression. (a) Workfunction of ITO measured by Kelvin probe. **(b)** Dark current density of the fabricated sensors at -2 V as a function of PEIE concentration in deionized water. Standard deviations are indicated in brackets.

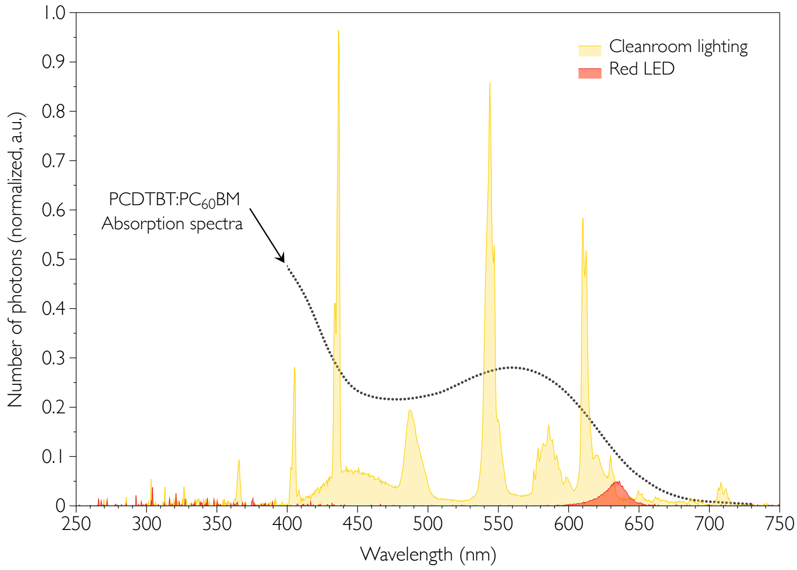

Supplementary Fig. 4 | Spectrometer measurements. To minimize the effect of photo-oxidation when screen-printing, OPDs were processed under extremely weak (< 50 lumens) monochromatic red light (636 nm), rather than the cleanroom lighting, taking advantage of low absorption of PCDTBT in the 625-700 nm region.

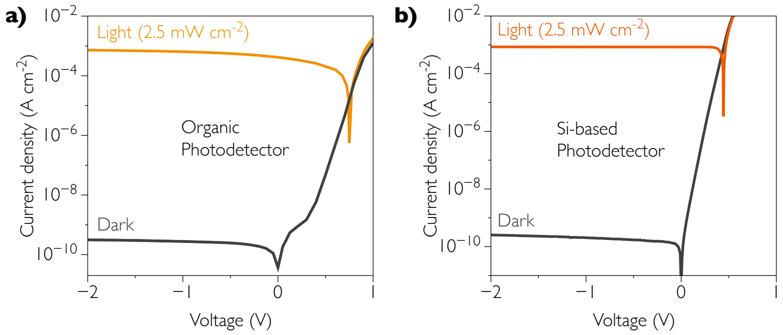

Supplementary Fig. 5 | Organic vs. inorganic photodetector. Current-voltage characteristics of **(a)** the optimized OPD and **(b)** Si photodetector (*Centronic Ltd*) under 528 nm light. The intensity of monochromatic light is set to 2.5 mW cm^{-2}.

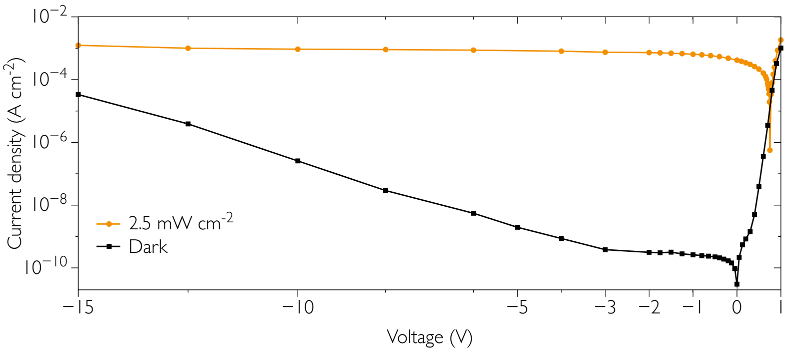

Supplementary Fig. 6 | Current-voltage characteristics from -15 to +1 V of the optimized device under 528 nm green light. The intensity of monochromatic light is set to 2.5 mW cm^{-2}. The responsivities are 0.268, 0.302 and 0.311 A W-1 for -1, -2 and -3 V respectively. The dark currents are 0.26, 0.31 and 0.38 nA cm^{-2} for -1, -2 and -3 V respectively. -3 V seems to be a critical voltage at which dark current start to increase rapidly.

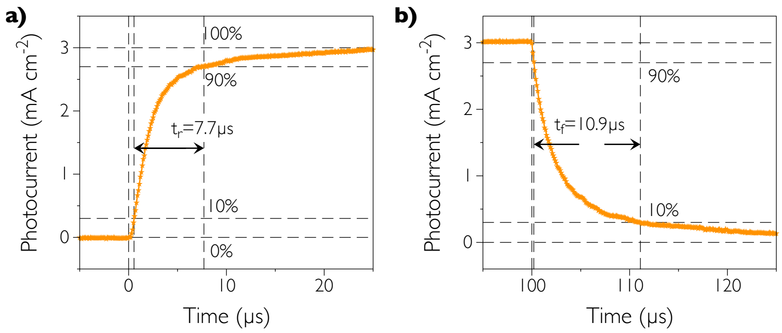

Supplementary Fig. 7 │ Transient photocurrent response. (a) Rise time (7.7×10^{-6} s) and **(b)** fall time (10.9×10^{-6} s) measurements for the optimized OPD.

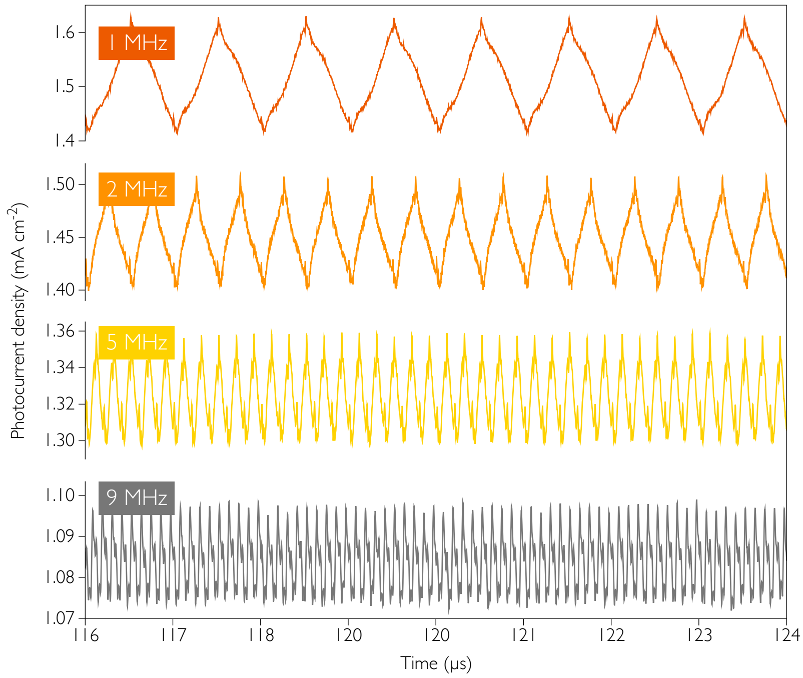

Supplementary Fig. 8 │ Transient photocurrent response at high frequencies. Photodetector response to the light pulse at ultra-high frequencies. The signal, even if low, is easily detectable.

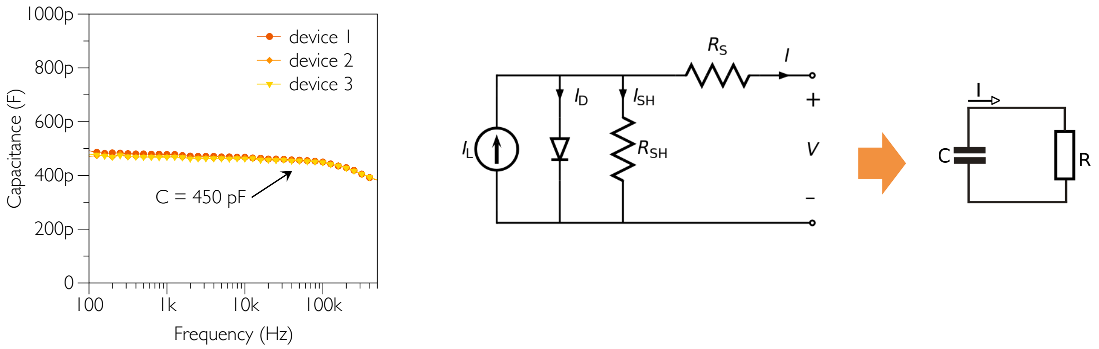

Supplementary Fig. 9 │ Equivalent RC circuit of the photodetector. Photodetector capacitance (450 pF) as a function of frequency (left) and simplified equivalent circuit of the photodetector (right). The cut-off frequency can be calculated from equation (1):

$$f_{cut-off} = \frac{1}{2\pi RC} \tag{1}$$

where R is the series resistance of the photodetector taken from the I-V curve (3.7 kΩ) and C is the capacitance (450 pF). The equivalent cut-off frequency is 95 kHz.

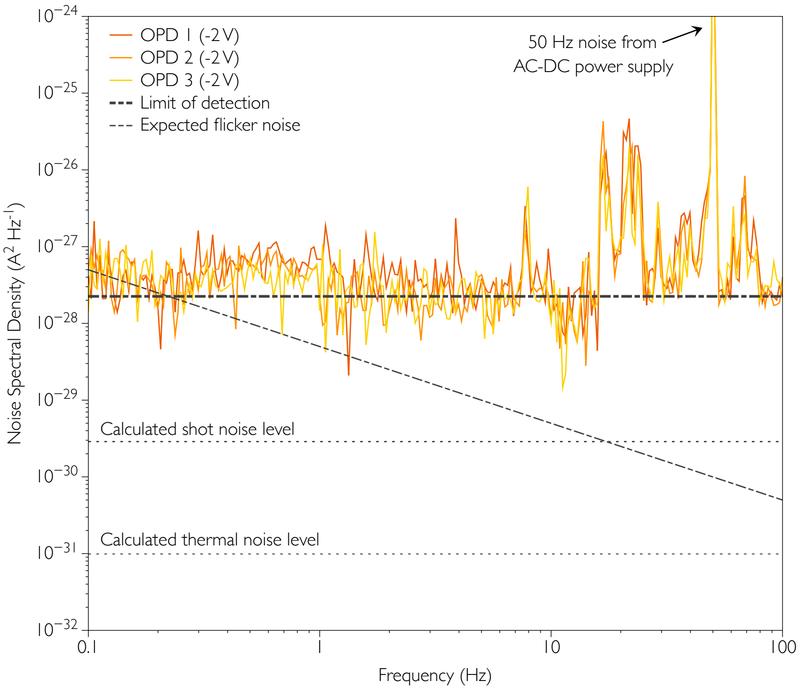

Supplementary Fig. 10 │ Noise spectral density (S_I) measurements for three organic photodetectors having dark current levels below 10 pA (< 0.36 nA cm^{-2}) at -2 V. No flicker noise is observed since its level is below the sensitivity of the measurement setup. The shot noise and thermal noise are calculated by using the equation (2) and (3)

$$S_{IS} = (I_s)^2 = 2qI_d \tag{2}$$

$$S_{ITH} = (I_{th})^2 = \frac{4kT}{R_{sh}} \tag{3}$$

where I_S is shot noise, I_{TH} is thermal noise, I_d is dark current (A), q is electron charge (C), k is Boltzmann's constant (J K^{-1}), T is absolute temperature (K) and R_{sh} is photodiode shunt resistance (Ω). The allure of the flicker noise is represented with the common assumption that the shot noise is dominant at frequencies higher than 20 Hz.

Supplementary Table 1. Sheet resistance of PEDOT:PSS measured with 4-Probe Unit. Standard deviations of the data are given in brackets.

	Current	Voltage	Sheet resistance
1	0.5	2.97 (± 0.11)	26.92 (± 0.98)
2	0.5	2.43 (± 0.13)	22.03 (± 1.18)
3	0.5	2.57 (± 0.17)	23.30 (± 1.54)
4	0.5	2.37 (± 0.15)	21.48 (± 1.36)
5	0.5	2.55 (± 0.12)	23.12 (± 1.09)
